# Type 3 Thyroplasty for a Patient with Female-to-Male Gender Identity Disorder

**DOI:** 10.1155/2018/4280381

**Published:** 2018-04-10

**Authors:** Yu Saito, Kazuhiro Nakamura, Shigeto Itani, Kiyoaki Tsukahara

**Affiliations:** ^1^Department of Otolaryngology, Head and Neck Surgery, Tokyo Medical University, 6-7-1 Nisisinnjuku, Shinjuku-ku, Tokyo 160-0023, Japan; ^2^Department of Otolaryngology, Todachuo General Hospital, 1-19-3 Honthou, Toda-shi, Saitama 335-0023, Japan

## Abstract

**Objective:**

In most cases, about the voice of the patient with female-to-male/gender identity disorder (FTM/GID), hormone therapy makes the voice low-pitched. In success cases, there is no need for phonosurgery. However, hormone therapy is not effective in some cases. We perform type 3 thyroplasty in these cases.

**Method:**

Hormone therapy was started in 2008 but did not lower the speaking fundamental frequencies (SFFs). We therefore performed TP3 under local anesthesia.

**Results:**

In our case, the SFF at the first visit was 146 Hz. The postoperative SFF was 110 Hz.

**Conclusions:**

TP3 was performed under local anesthesia in a patient with FTM/GID in whom hormone therapy proved ineffective. With successful conversion to a lower-pitched voice, the patient could begin to live daily life as a male. QOL improved significantly with TP3. If hormone therapy proves ineffective, TP3 may be selected as an optional treatment and appears to show few surgical complications and was, in this case, a very effective treatment.

## 1. Introduction

Gender identity disorder is a state in which self-consciousness of sex and biological sex is different. Treatments for female-to-male/gender identity disorder (FTM/GID) include hormone therapy, plastic surgery, sex reassignment surgery, voice therapy, and phonosurgery. In most cases, hormone therapy results in the voice of patients with FTM/GID becoming low-pitched. In successful cases, no phonosurgery is needed. However, hormone therapy is ineffective in some cases, and we perform type 3 thyroplasty (TP3) for such cases. TP3 is a surgical technique used to reduce the anteroposterior diameter of the thyroid cartilage. This causes the vocal folds to shorten and relax. With the decrease in tension of the vocal fold, speaking fundamental frequencies (SFFs) decrease and the voice becomes lower-pitched. The surgery is also effective for improving laryngeal efficiency. TP3 is effective for diseases such as vocal fold atrophy, sulcus vocalis, mutation voice disorder, and scarring.

## 2. Patient and Methods

**Figure 1:**
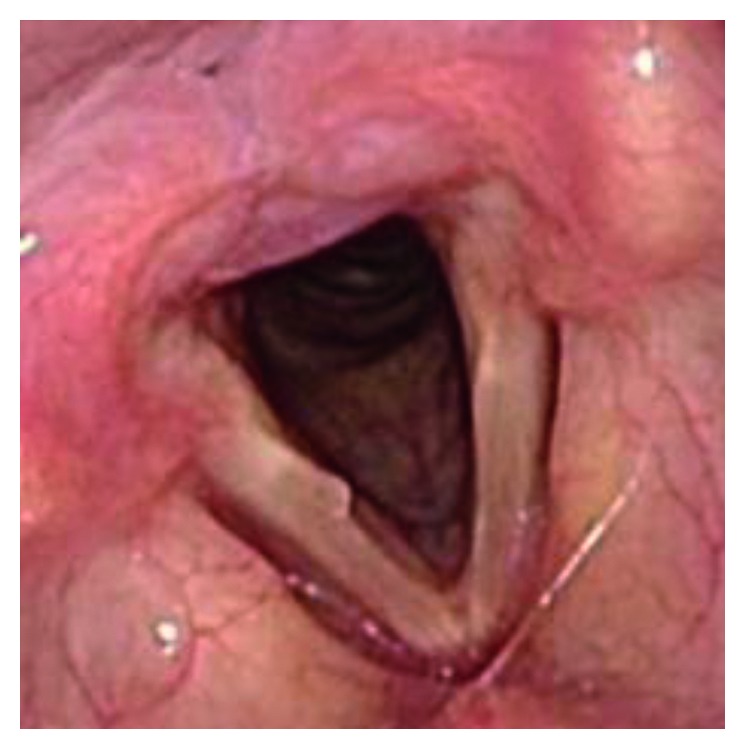
Preoperative endoscopic findings in aspiration.

**Figure 2:**
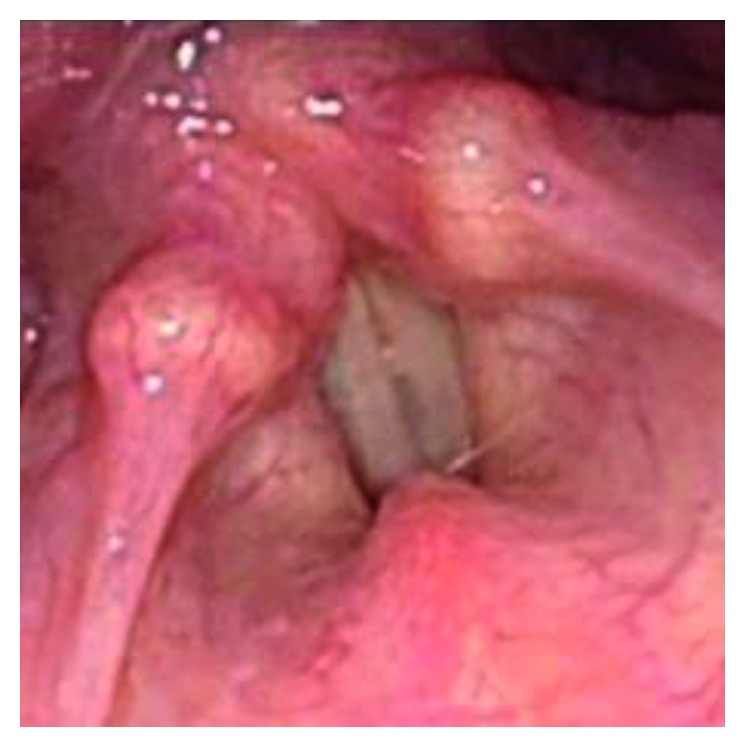
Preoperative endoscopic findings in phonation.

The patient was a 46-year-old individual with FTM/GID, before sex reassignment surgery (SRS). The chief complaint was a high-pitched voice. In terms of past history, she had a history of appendicitis and panic disorder. The subject became aware of GID around 4 years old. FTM/GID was diagnosed at the age of 42 years. Hormone therapy was started in 2008 but did not lower the SFF, and Kayser-Gutzmann voice therapy was ineffective. She came to our hospital for phonosurgery in August 2015. Figures 1 and 2 showed preoperative endoscopic findings in aspiration and phonation. SFF at the first visit was 146 Hz, and no abnormalities of the vocal folds were identified. To measure the F0, the patient was instructed to phonate the sustained vowel /e/ with a comfortable tone and loudness. Hormone therapy and speech therapy failed to sufficiently lower the pitch of the voice. We therefore selected TP3 under local anesthesia for this case.

## 3. Operation Methods and Results

**Figure 3:**
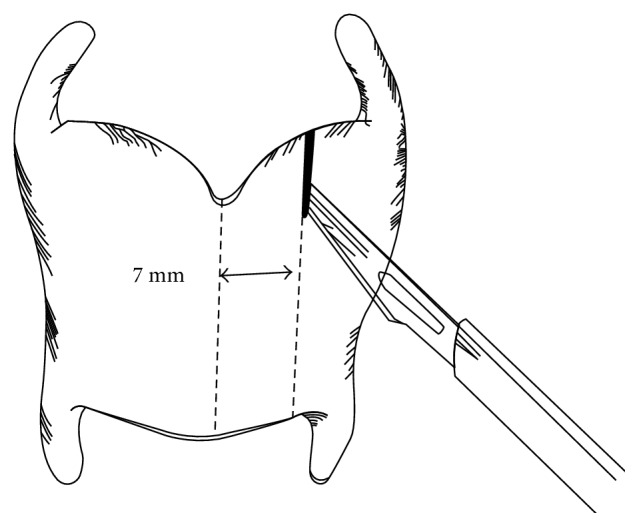
Cartilage incision. An incision with a number 11 scalpel is made on the lateral side of the thyroid cartilage.

**Figure 4:**
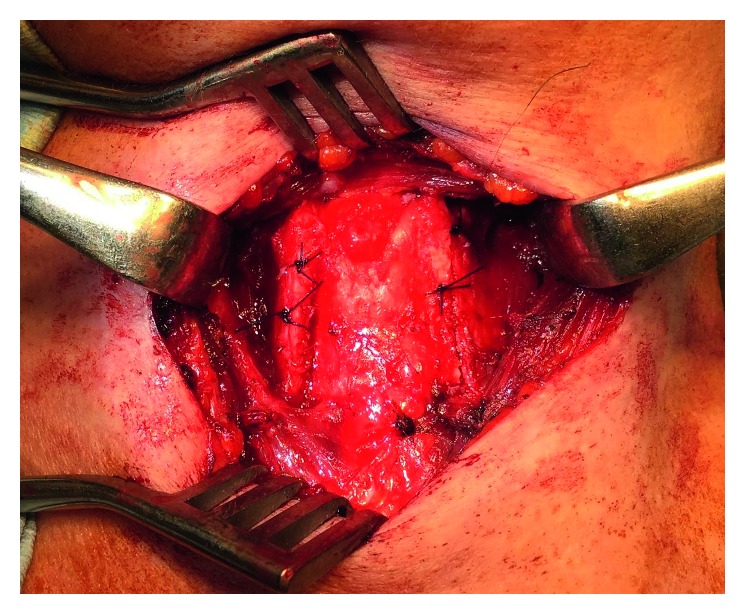
Findings of thyroid cartilage at the end of surgery. The right was overlapped, and the left side was returned and fixed FTF.

**Figure 5:**
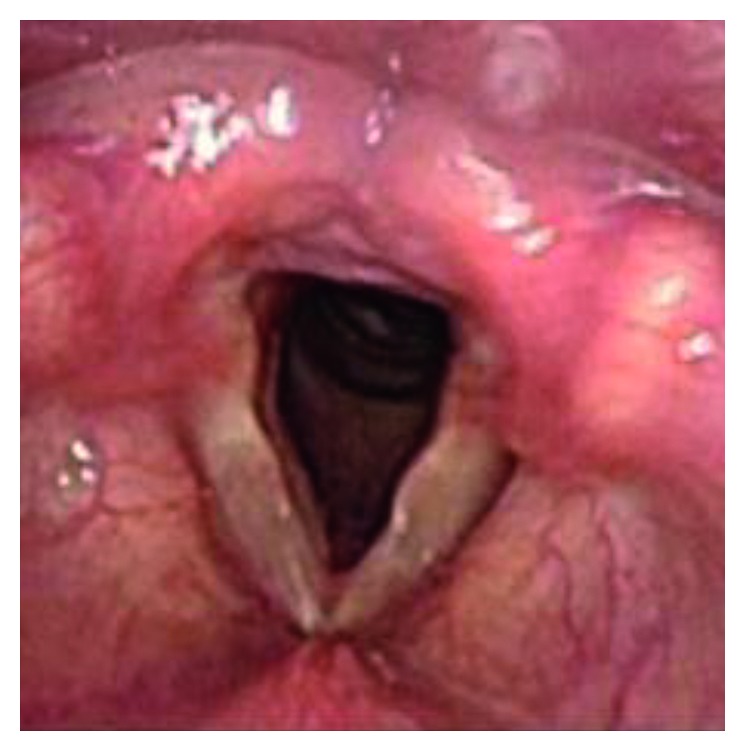
Postoperative endoscopic findings in aspiration.

**Figure 6:**
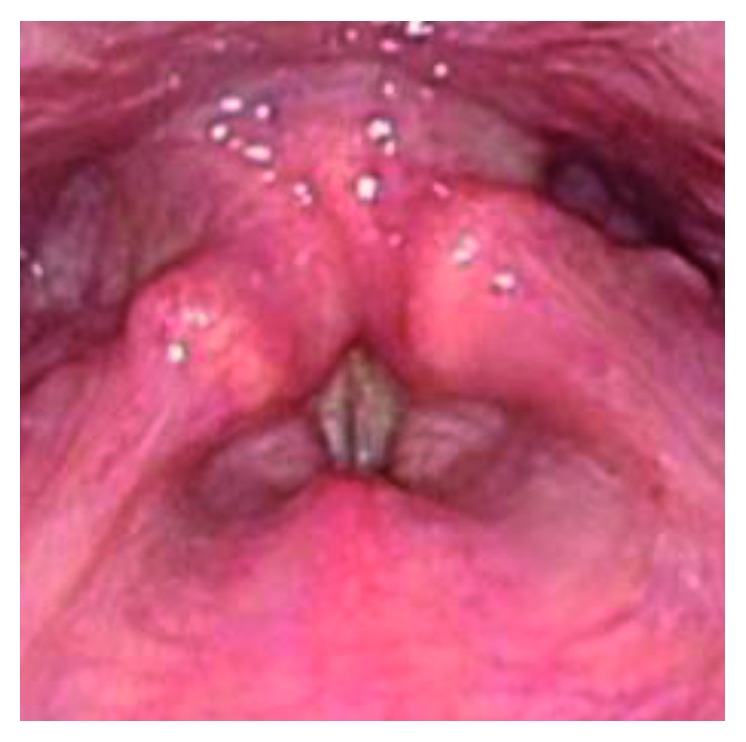
Postoperative endoscopic findings in phonation.

The surgery was performed under local anesthesia to allow the voice to be monitored. A horizontal skin incision was made in the neck. A vertical incision was made to separate and retract the strap muscles laterally and expose the thyroid ala on one side. An incision about 7 mm long is then made with a number 11 scalpel on the lateral side of the thyroid cartilage (Figure 3). TP3 was started from one side according to the original Isshiki method. After splitting the thyroid cartilage on the right side, the cartilage was overlapped. The voice then became markedly lower-pitched. Next, the thyroid cartilage was split on the left side and overlapped. At this time, the voice turned rough and spasmodic. Finally, overlapping up to a width of 3 mm was carried out on the right side, while the left side was returned and fixed face-to-face (FTF). Fixation is important. The thyroid cartilage was fixed by a 3-0 nylon suture and a 17 mm curved conventional cutting needle. Further monitoring showed the voice was not spasmodic and remained low-pitched. We confirmed that the patient was satisfied with her voice and the operation was finished (Figure 4). The operation lasted 1 h 35 min. Postoperative SFF was 110 Hz, and quality of life (QOL) improved significantly with TP3 (Table 1). The Jitter and Shimmer score was not changed from preoperation to postoperation (Table 1). Figures 5 and 6 showed postoperative endoscopic findings in aspiration and phonation.

## 4. Discussion

In the treatment of FTM/GID, hormone therapy makes the voice low-pitched in most cases. As a result, phonosurgery is not needed. In hormone therapy, an androgen or anabolic steroid is administered, and there are few side effects [1]. SFF becomes low-pitched within 1-2 months, stabilizing at 6 months. However, predicting how much vocal degradation will be seen is difficult, given the individual differences [2]. In voice therapy, the Kayser-Gutzmann method is often performed and usually effective in mutation voice disorders. In this case, hormone therapy and voice therapy failed to sufficiently lower the pitch of the voice. TP3 under local anesthesia was selected for this Case [3–6]. TP3 was first reported by Isshiki et al. in 1974 [7], with further descriptions by the same group in 1983 [3]. TP3 surgery involves relaxation of the vocal folds. Partial resection of the thyroid cartilage causes the vocal folds to shorten and relax, and with decreased tension of the vocal folds, the SFF decreases and the voice becomes lower-pitched [4–6]. During TP3, monitoring the voice is very important. TP3 should therefore be performed under local anesthesia, not general anesthesia, and we continued surgery while confirming the patient's voice. In this manner, it is possible to choose the voice the patient wants, and the patient can also be satisfied [8].

**Figure 7:**
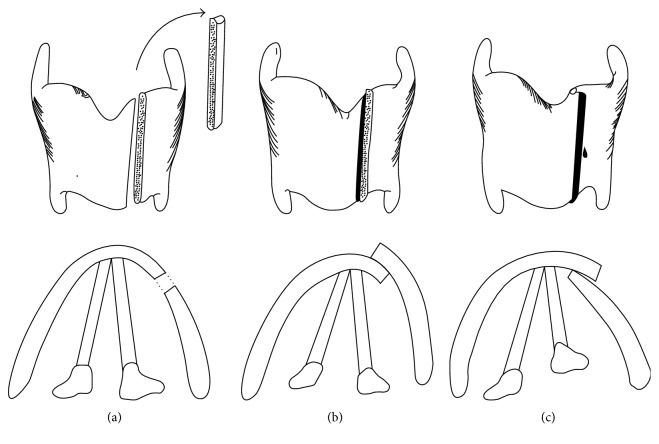
Three fixation patterns (a) FTF fixation. (b) OL fixation: lateralization. (c) OL fixation: medialization.

Under voice monitoring, the edges may also be overlapped, if necessary. There are two kinds of OL: medialization and lateralization (Figure 7). Medialization or lateralization and the OL width are determined under voice monitoring. In this case, firstly, the voice turned good with lateralization OL, the right side was fixed in a lateralization OL manner. Secondly, the thyroid cartilage was split on the left side and overlapped, the voice was did not turn out to be good. Whereas the left side was returned and fixed in a face-to-face (FTF) manner as before. The major indications include patients with a high SFF associated with vocal fold atrophy, mutational voice disorder, FTM/GID, or laryngeal trauma, and patients with sulcus vocalis, who show unsatisfactory responses to voice therapy [4, 5, 9, 10]. In either case, tension decreases due to shortening of the anteroposterior diameter of the vocal fold and SFF decreases, leading to vocal improvement. Unlike other thyroplasties, TP3 is irreversible surgery because it involves cartilage resection in cases of fixed FTF. Appropriate preoperative evaluation of the patient is thus important. The AP test is the most important preoperative exam. We confirm whether the SFF is low by pressing the thyroid cartilage from the ventral side to the dorsal side.

In our case, the VHI score was decreased slightly, and the QOL improved. In Japan, many patients with GID stand miserable situations. Many Japanese have prejudice and discrimination against GID. His preoperative voice is almost normal as shown in Table 1, but he has been taking a lot of prejudices and discriminations against the high-pitched SFF. He looked real man, but his voice was high-pitched, he has been worried about his voice long time. So, he came to us to take TP3. TP3 could change his voice low-pitched; he was reborn as real man. His QOL improved by TP3.

## 5. Conclusion

TP3 was performed in a patient with FTM/GID in whom hormone therapy proved ineffective. With successful conversion to a lower-pitched voice, the patient could begin to live daily life as a male. QOL improved significantly with TP3, which was performed under local anesthesia. Voice characteristics can be adjusted to some extent and patient feedback improves satisfaction with the operation. If hormone therapy proves ineffective, TP3 may be selected as optional treatment.

## Figures and Tables

**Table 1:** Pitch range, acoustic analyzed data and VHI of pre- and post-operative voice.

	Lowest (Hz)	SFF (Hz)	Highest (Hz)	Jitter (%)	Shimmer (%)	VHI-30
Preope	136	146	493	0.54	1.01	9/120
Post-ope	92	110	402	0.91	1.82	4/120
